# Peripheral Arterial Disease and Its Associated Factors among Type 2 Diabetes Mellitus Patients at Debre Tabor General Hospital, Northwest Ethiopia

**DOI:** 10.1155/2020/9419413

**Published:** 2020-01-29

**Authors:** Yonas Akalu, Ambaye Birhan

**Affiliations:** ^1^Department of Physiology, College of Medicine and Health Sciences, University of Gondar, Gondar, Ethiopia; ^2^Department of Radiology, College of Medicine and Health Sciences, Debre Tabor University, Debre Tabor, Ethiopia

## Abstract

**Background:**

Peripheral arterial disease (PAD), one of the major macrovascular complications of diabetes, is associated with cardiovascular mortality and a high rate of disability following the amputation of an extremity in diabetes patients. However, there is no data on the prevalence of PAD among type 2 diabetes patients in Ethiopia. Therefore, the current study is aimed at determining the prevalence and associated factors of PAD among type 2 diabetes patients at Debre Tabor General Hospital, Debre Tabor, Northwest Ethiopia.

**Methods:**

An institution-based cross-sectional study was conducted among type 2 diabetes mellitus patients from February 1 to August 30, 2019. A pretested interviewer-administered questionnaire was used to collect the data. The presence of stenosis and its grading were determined by color Doppler ultrasonography. Data were entered using EpiData-V.4.6 and analyzed by STATA-14. Bivariable and multivariable logistic regression was performed to identify associated factors of peripheral arterial disease. Adjusted odds ratio (AOR) and its confidence interval were estimated for potential predictors included in the final model. *P* ≤ 0.05 was used to declare statistical significance.

**Results:**

The mean age of the study participants was 61.2 ± 7.3 years. One hundred seventy-two (61.4%) patients were males. The prevalence of PAD in this study was 30.7% (95% CI (25.3-36.2%)). Of these, 37 (43%) were symptomatic. Age (AOR = 1.09, 95% CI (1.03-1.16)), higher HbA1c (AOR = 1.97, 95% CI (1.03-3.40)), being an ex-smoker (AOR = 4.68, 95% CI (1.93-11.30)), and current cigarette smoking (AOR = 5.84, 95% CI (1.79-19.04)) were significantly associated with PAD.

**Conclusion:**

The prevalence of peripheral arterial disease among type 2 diabetes patients was high. Increasing age, high HbA1c, and being cigarette smokers increase the likelihood of developing peripheral arterial disease. Clinicians should prevent PAD; screen T2DM patients who are aged, with high HbA1c, and cigarette smokers; and treat them timely.

## 1. Background

Diabetes is a complex chronic metabolic disorder, requiring continuous medical care with multifactorial risk reduction strategies, characterized by persistent hyperglycemia because of lack of insulin secretion, insulin resistance, or both [1, 2]. Its prevalence is steadily increasing in the world, most markedly in the lower- and middle-income countries [2, 3] like Ethiopia, which is the first among the top five countries of Africa for a number of people with diabetes [4]. Through time, T2DM leads to early microcomplications, peripheral neuropathy, peripheral retinopathy, and peripheral nephropathy, and late macrocomplications, which are a consequence of atherosclerosis of the arteries, including peripheral arterial disease, coronary artery disease, and cerebrovascular accident which all are potentially life-threatening [5, 6]. Among these, peripheral arterial disease (PAD) is one of the major complications of diabetes. Peripheral arterial disease is defined as an atherosclerotic narrowing of peripheral arteries of the legs, stomach, arms, and the head—most commonly involving arteries of lower extremities [7, 8]. It is a major complication of atherosclerosis [8] as well as a manifestation of atherosclerosis in major blood vessels like coronary and cerebral arteries [9, 10]. It results in systemic atherothrombosis that leads to cerebrovascular events, including myocardial infarction, stroke, significant long-term disability, and death [9, 11–13]. Diabetic patients with PAD are at high risk of increased morbidity and mortality from cardiovascular diseases and a high rate of lower extremity amputation [7, 14–16]. This increased risk of amputation in diabetes patients is due to dry gangrene [17], end-stage presentation of PAD, and foot ulcer secondary to PAD [18]. Almost two-thirds of diabetic patients with foot ulcers have PAD, which is associated with a high amputation rate and mortality [19]. The prevalence of PAD is 3 to 4 times higher and severe in diabetic individuals compared with nondiabetic individuals [11, 20–22]. The global prevalence of PAD is estimated to be 202 million [23, 24]. Twelve percent of the adult population has PAD [25]. A study in Korea revealed that the prevalence of PAD among type two diabetic patients was 28.7% [16]. There was a 36% prevalence of PAD among DM patients in India [11] whereas it was 24% in Uganda [8]. A prevalence of 22% was reported in Nigeria diabetic patients [26]. Age, gender, duration of diabetes mellitus, persistent hyperglycemia, increased glycated hemoglobin (HbA1c), smoking, hypertension, dyslipidemia, and obesity are associated with an increased risk of PAD [9, 17, 27]. Among these, the most common risk factors associated with PAD are increasing age, diabetes, and smoking [26, 28, 29]. Early diagnosis and treatment of PAD in a diabetes patient are critically important for risk factor modification, reduction of its prevalence, progression and improvement of its outcome [17], improving quality of life, preventing cardiovascular events, and minimizing the risk of long-term disability and other complications associated with it [16, 30, 31]. However, most patients with PAD are asymptomatic and did not complain intermittent claudication due to decreased pain perception secondary to peripheral neuropathy [32, 33]. Of those who are symptomatic, only a small proportion of the diabetic population reports it due to a lack of awareness about symptoms of PAD [29]. This, in turn, delays the recognition and diagnosis of PAD [32]. These asymptomatic nature, lack of awareness, and underutilization of screening tools made PAD underestimated and untreated [16]. Even though diabetes is highly prevalent in Ethiopia, and results in major complications like PAD, nothing is known about the prevalence and associated factors of PAD among diabetes patients in Ethiopia. Therefore, this study is aimed at filling this gap by determining the prevalence and associated factors of PAD among T2DM patients at Debre Tabor General Hospital, Ethiopia. Furthermore, this study adds information to the scientific community and will serve as a baseline for future researchers.

## 2. Methods and Materials

### 2.1. Study Area and Period

An institution-based cross-sectional study was conducted from the 1^st^ of February to the 30^th^ of August 2019 at Debre Tabor General Hospital in Debre Tabor town, which is located 667 km to the northwest of Addis Ababa, the capital city of Ethiopia.

### 2.2. Source and Study Population

All diabetes mellitus patients who were attending the diabetic follow-up clinic at Debre Tabor General Hospital were our source of population. This study included all type 2 diabetes patients aged ≥ 50 years who attended the diabetic follow-up clinic at Debre Tabor General Hospital during the study period.

### 2.3. Inclusion and Exclusion Criteria

All T2DM patients aged ≥ 50 years present during the study period were included. But T2DM patients who were severely ill; patients with lower limb swelling due to filariasis, inflammation, or other causes, which would impair the Doppler image quality; patients with a history of cardiovascular disease (CVD) including stroke; and patients with a history of hypertension before a diagnosis of DM were excluded from the study.

### 2.4. Sample Size and Sampling Procedure

The required sample size for the study was estimated using a single population proportion formula by taking a 24% proportion of PAD among diabetic patients of Uganda [8], 0.05 level of significance (*α*), and 5% margin of error. Accordingly, the final sample size for this study was 280. We consecutively enrolled a total of 280 type two diabetic patients.

### 2.5. Data Collection Procedure

A pretested, interviewer-administered questionnaire was used to collect data. The questionnaire was adapted from previously published articles [11, 13, 16, 17, 25, 34–36], prepared in English and translated to Amharic, and retranslated to English by another person. To assess symptoms of PAD, intermittent claudication, we administered a validated Edinburgh Claudication Questionnaire (ECQ) [37]. Three data collectors and one supervisor participated in data collection. Data were also extracted from patient charts. Anthropometric measurements were taken using standardized techniques and calibrated equipment. Body weight (kg) and height (cm) were measured to the nearest 0.1 cm and 0.1 kg, respectively, in barefoot subjects wearing light clothing. Body mass index (BMI) was calculated as weight divided by height squared (kg/m^2^). Blood pressure (BP) was recorded using a mercury sphygmomanometer BP cuff with the appropriate size that covers two-thirds of the arm with the subject in the sitting position. The arm which was used for BP measurement was supported on a flat table. Participants were asked to rest for at least 5 minutes, and if they were taking any caffeinated beverages, they were rested for 30 minutes before measurement. Two consecutive measurements were taken 5 minutes apart, and the mean value was used. HbA1c was measured using a 902 Automatic Analyzer with a Roche/Hitachi kit (Roach Diagnostic, USA).

#### 2.5.1. Diagnosis of PAD

Diagnosis of PAD was made by using color Doppler ultrasound with sensitivity of 97%, specificity of 81%, and diagnostic accuracy of 85% [38]. To eliminate interobserver variation, a single senior radiologist performed all Doppler studies using color Doppler ultrasonography (Sonoscape SSI 8000). Grading of stenosis was made according to Jager's criteria.

### 2.6. Operational Definitions

PAD was defined as Grade III (50% to 99% stenosis) or IV stenosis (100% stenosis) by color Doppler ultrasonography [16]. Grade I is 1% to 19% stenosis: normal triphasic flow with normal peak systolic velocity with spectral broadening. Grade II is 20% to 49% stenosis: a triphasic waveform with an increase in peak systolic velocity = 30% concerning the proximal recording site, marked spectral broadening. Grade III is 50% to 99% stenosis: a monophasic waveform with an increase in peak systolic velocity = 100% and marked spectral broadening. The distal waveform is abnormal. Grade IV is 100% stenosis: no forward flow detected with altered flow patterns, both proximal and distal to the stenosis [39]. Hypertension (HTN) is systolic blood pressure (SBP) and/or Diastolic Blood Pressure (DBP) of 140/90 mmHg or greater [40]. Typical claudication was defined as the presence of calf pain, regardless of whether there was a pain at other sites. Atypical claudication was defined as having pain in the thigh or buttock in the absence of calf pain. No claudication was defined as having pain in the hamstrings, feet, shins, or joints; or any calf, thigh, or buttock pain that appears to radiate; or no pain at all in any part of the leg [37]. For body mass index, a person with a BMI of 18.5–24.9 kg/m^2^, 25–30 kg/m^2^, and >30 kg/m^2^ is considered as normal, overweight, and obese, respectively. Ex-smokers were participants who did not currently smoke but had a history of cigarette smoking in their lifetime. Current smokers were defined as participants who smoked a cigarette at least once in the last month before the study.

### 2.7. Data Quality Control

To assure data quality, first, the questioner was pretested on 14 type 2 diabetes mellitus patients at another diabetic clinic (at Addis Zemen District Hospital). Data was collected under regular supervision after giving training for data collectors. Data were also properly entered and coded before analysis.

### 2.8. Data Processing and Analysis

Data were entered using EpiData-V.4.6 and analyzed by STATA-14. We expressed continuous data by the mean ± standard deviation and categorical variables by percentages. The Chi-squared test was used, after checking its assumption, to examine the difference between categorical variables. Both binary and multivariable logistic regression analyses were performed to identify risk factors for PAD. Variables in bivariable analysis with *P* < 0.2 were entered into multivariable logistic regression. In multivariable logistic regression, variables with *P* ≤ 0.05 were declared statistically significant. Goodness of fit of the statistical model was checked by the Hosmer-Lemeshow test (*P* = 0.065). Furthermore, interaction of independent variables that were a candidate for the multivariable logistic regression model was checked. The strength of the association of risk factors with PAD was demonstrated by computing the crude odds ratio (COR) and the adjusted odds ratio (AOR) with a 95% confidence interval (CI).

### 2.9. Ethical Consideration

Ethical approval for the study was obtained from the Institute of Public Health College of Medicine and Health Sciences, University of Gondar. Written informed consent was obtained from all study participants, and confidentiality was kept. All the study subjects had answered the administered pretested questionnaires voluntarily and confidently.

## 3. Results

### 3.1. Sociodemographic and Clinical Characteristics of Study Participants

The study was conducted on 280 T2DM patients. The mean age of the study participants was 61.2 ± 7.3, ranging from 50 to 91 years. One hundred seventy-two (61.4%) patients were male. The majority (70.4%) of study participants live in an urban area. Regarding educational status, 97 (34.6%) study participants cannot read and write. Ninety-three (33.2%) study participants were a governmental employee. Half of the study participants were diabetic for 10-20 years. The majority (73.2%) of the study participants were overweight. Two hundred fifteen (76.8%) respondents use an oral hypoglycemic agent as a treatment option for T2DM. The mean HbA1c of respondents was 6.9 ± 1.13. Thirty-nine (13.9%) and twenty-nine (7.5%) respondents were ex-smoker and current cigarette smokers, respectively, while the remaining are nonsmokers. Regarding hypertension status, 160 (57.1%) respondents were currently hypertensive, while 146 (52.1%) had history of hypertension (Table 1).

### 3.2. Prevalence of PAD and Degree of Stenosis among T2DM Patients

**Figure 1:**
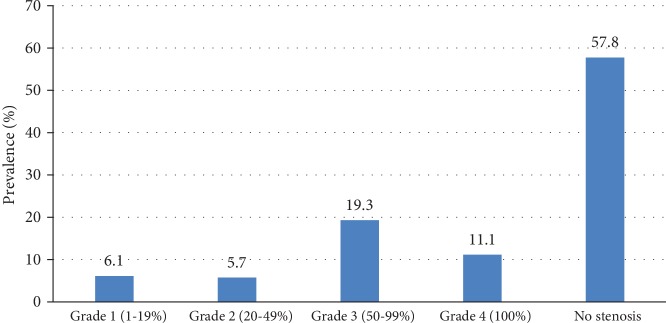
Degree of stenosis on color Doppler ultrasonography in patients with T2DM. Grade III and IV stenoses were defined as peripheral artery disease.

The prevalence of PAD was 30.7% (95% CI (25.3-36.2%)); among these, 37 (43%) were symptomatic. Of those who were symptomatic, 30 (81.1%) had typical intermittent claudication (Table 2). Regarding the degree of stenosis, 162 (27.8%) T2DM patients had no stenosis while the other 118 (43.2%) have a different degree of stenosis (Figure 1).

### 3.3. Factors Associated with PAD among T2DM Patients

Crude association of all independent variables with the dependent variable PAD was checked by binary logistic regression. Accordingly, age, educational status, DM duration, BMI, type of antidiabetic drug on use, HbA1c, cigarette smoking, history of hypertension (HTN), and current HTN were a candidate for the final model. After adjusting for potential confounders in the multivariable analysis, age, HbA1c, and cigarette smoking were significantly associated with PAD. A 1-year increase in age of DM patients was associated with a 9% higher odds (AOR = 1.09, 95% CI (1.03-1.16)) of PAD. Each 1% increase in HbA1c was associated with 1.9 times (AOR = 1.97, 95% CI (1.03-3.40)) higher odds of developing PAD.

The odds of PAD among T2DM patients were 4.7 times higher (AOR = 4.68, 95% CI (1.93-11.30)) in ex-smokers than nonsmokers. Similarly, the odds of PAD among T2DM patients were 5.8 times (AOR = 5.84, 95% CI (1.79-19.04)) higher in current smokers than nonsmokers (Table 3).

## 4. Discussion

A total of 250 diabetic patients were included in the study. Color Doppler ultrasound-proven prevalence of PAD among type 2 diabetics was 30.7%.

This high prevalence is a result of hyperglycemia, dyslipidemia, and insulin resistance, secondary to DM, which all induce development and progression of PAD or atherosclerosis by disrupting the vessel wall through promotion of vascular inflammation and endothelial cell dysfunction, derangements of various cell types like platelets within the vascular wall, promotion of coagulation, and inhibition of fibrinolysis [41]. This finding is in line with a finding of a study done in the United States (29%) [42], Korea (28.7%) [16], Uttar Pradesh, India (36%) [11], India (35%) [43], and Pakistan (31.6%) [43].

Nonetheless, the prevalence of PAD in the current study is higher than the result of a study done in Uganda (24%) [8], Nigeria (22%) [26], Coastal Karnataka, India (8.5%) [13], and South India (16.5%) [13, 20]. The possible reason for this difference might be due to a difference in the type of diagnostic tool used. Color Doppler ultrasound was used in the current study which is the best diagnostic technique compared with other diagnostic methods like the ankle-brachial index, which can underestimate the prevalence of PAD [16]. Another possible cause of difference may be a variation in the age of study participants, drug adherence, and management of T2DM. On the other hand, the prevalence of PAD in the current study is lower than that of the study done in Uganda (39%) [44] and Pakistan (39.3%) [45]. This might be due to a difference in socioeconomic factors, lifestyle, and duration of diabetes of study participants.

In the current study, age, HbA1c, being an ex-smoker, and current cigarette smoking were significantly associated with PAD. Each 1-year increase in age of DM patients was associated with 9% higher odds of developing PAD. This is supported by a study done in Korea [16], Nigeria [26], Greece [17], Boston, United States [36], and India [11, 46]. The reason for an increase in the prevalence of PAD with an increase in age is due to the reason that with age, each layer of blood vessels changes in complex ways and triggers arterial stiffening and thickening. Thickening of the intima due to aging compromises endothelium integrity and decreases the availability of nitric oxide, a known vasodilator. Stiffening of the arterial walls disturbs the normal blood flow that makes it easier for calcium and fatty deposits to build upon the inside of arteries which leads to further fatty build-up and narrowing of the vessel resulting in PAD [47–49].

Each 1% increase in HbA1c was associated with 1.9 times higher odds of developing PAD. This finding was consistent with the findings of other studies [17, 50, 51]. This is because of the effect of the process of glycation. Since HbA1c is a compound synthesized from the nonenzymatic glycation reaction of glucose and hemoglobin, especially in poorly controlled diabetes [52], an increase in HbA1c is associated with increased glycation, nonenzymatic addition of glucose to amino groups of proteins, due to hyperglycemia. Glycation enhances the covalent binding of lipoproteins to vascular wall proteins, promoting sequestration, free radical release, and hence inducing inflammation which all lead to the development of atherosclerosis and PAD. Antithrombin III may also be glycated resulting in impairment of its function and increase in thrombotic tendency that in turn triggers atherosclerosis [53, 54]. Regarding cigarette smoking, the current study showed that the odds of PAD among T2DM patients were 5.8 and 4.7 times higher in the current smoker and ex-smokers, respectively, than nonsmokers. Consistently, a study done in the United States revealed that current smokers have 4 times more risk of PAD than that of nonsmokers [55]. Similarly, a study done in the United Kingdom showed that cigarette smoking can result in a sevenfold increase in the risk of peripheral arterial disease [56]. A study done in Korea [16] also revealed a similar result. The reason why smokers are more prone to PAD is due to the toxic effect of nicotine, carbon monoxide, and other ingredients in it on blood vessels. Nicotine impairs endothelium-dependent vasodilatation by reducing nitric oxide production [57]. Nicotine stimulates the release of catecholamine, which increases platelet aggregability and thrombosis. Platelets contribute to the growth of plaque through the accretion of thrombus. Nicotine also induces insulin resistance and dyslipidemia, vascular inflammation, abnormal vascular growth, and loss of endothelial homeostatic and regenerative functions. Also, nicotine has direct actions on the cellular elements participating in plaque formation. All these effects of nicotine predispose to peripheral arterial disease [58], as well as through the release of growth factors (such as platelet-derived relaxing factor) that induce vascular smooth muscle cell proliferation. Moreover, nicotine has direct actions on the cellular elements participating in plaque formation to increase plaque progression independently of plasma lipid values [59, 60]. In addition to nicotine, free radicals directly from the component of cigarette smoke or activation of endogenous source free radicals lead to oxidative stress which is a major contributor to atherosclerosis and peripheral arterial disease [61].

In this study, sex, current high blood pressure, duration of DM, type of antidiabetic drug in use, and BMI were not significantly associated with PAD. This is in contrary to other studies which showed that female sex [8, 20, 62], current high blood pressure, being on a sulfonylurea-glibenclamide [8], duration of DM [20, 63], and BMI [36] are significantly associated with PAD. This disagreement might be due to a difference in characteristics of study population, sample size, drug adherence, and study design.

### 4.1. Limitation of the Study

This study had limitations that should be considered. Since the study participants were taken only from a single diabetes center, the findings may not represent that of the general diabetes population. Even though the use of antiplatelet agent drugs may affect vascular function, we were unable to find data about it. Despite these limitations, we determined PAD using color Doppler ultrasonography, which is the best imaging technique to detect PAD without any side effects.

## 5. Conclusion

The prevalence of PAD among diabetes patients was high. Age, HbA1c, and cigarette smoking were significantly associated with PAD. Clinicians should prevent PAD; screen all T2DM patients especially those aged, with high HbA1c, and cigarette smokers; and treat them timely. A large-scale study, involving lipid profile and other important laboratory assessments, should be done on multiple diabetic centers.

## Figures and Tables

**Table 1:** Sociodemographic and clinical characteristics of T2DM patients, Debre Tabor, Northwest Ethiopia, 2019.

Variable	Categories	Frequency/mean ± SD	Percent
Age (years)^c^		61.2 ± 7.3	

Sex	Male	172	61.4
	Female	108	38.6

Residence	Urban	197	70.4
	Rural	83	29.6

Educational status	Cannot read and write	97	34.6
	Read and write	57	20.4
	Elementary (1-8)	48	17.2
	High school and above	78	27.9

Occupation	Farmer	44	15.7
	Government employee	93	33.2
	Merchant	72	25.7
	Housewife	62	22.1
	Other^∗^	9	3.2

DM duration (year)	<10	119	42.5
	10-19	141	50.4
	≥20+	20	7.1

BMI (kg/m^2^)	Normal	18	6.4
	Overweight	205	73.2
	Obese	57	20.4

Type of antidiabetic drug	OH	215	76.8
	Insulin	21	7.5
	Both OH and insulin	44	15.7

HbA1c (%)^c^		6.9 ± 1.13	

Cigarette smoking	Nonsmoker	220	78.6
	Ex-smoker	39	13.9
	Current smoker	21	7.5

History of HTN self-reported	Yes	146	52.1
	No	134	47.9

Current hypertension (high blood pressure)	Yes	160	57.1
	No	120	42.9

Other^∗^ = daily laborer, nongovernmental organization workers; ^c^continuous variables expressed as the mean ± SD; OH = oral hypoglycemic agent.

**Table 2:** Comparison of PAD symptoms between PAD-positive and PAD-negative DM patients, Debre Tabor, Northwest Ethiopia, 2019.

Symptom status	Total	PAD (*N* = 86)	No PAD (*n* = 194)	Statistical significance	
		*N* (%)	*N* (%)	*χ* ^2^	*P* value
Asymptomatic	222 (79.3%)	49 (22.1)	173 (77.9)	37.6	*P* < 0.0001
Symptomatic	58 (20.7%)	37 (63.8)	21 (36.2)		
Typical symptom	35 (12.5%)	30 (85.7)	5 (14.3)	18.3	*P* < 0.0001
Atypical symptom	23 (8.0%)	7 (30.4)	16 (69.5)		

**Table 3:** Factors associated with PAD among type 2 diabetes mellitus patients in bivariable and multivariable logistic regression analyses, Debre Tabor, Northwest Ethiopia, 2019.

Variable	PAD		OR (95% CI)	
	Yes (*n* = 86)	No (*n* = 194)	COR	AOR
Age (year)^C^	64.7 ± 8.9	59.6 ± 5.8	1.11 (1.06-1.15)	1.09 (1.03-1.16)^∗∗^
Sex				
Male	57 (33.1)	115 (66.9)	1.35 (0.80-2.30)	
Female	29 (26.9)	79 (73.1)	1	
Residence				
Urban	63 (31.9)	134 (68.1)	1.23 (0.70-2.16)	
Rural	23 (27.7)	60 (72.3)	1	1
Educational status				
Cannot read and write	24 (24.7)	73 (75.3)	0.84 (0.43-1.65)	1.27 (0.56-2.95)
Read and write	18 (31.6)	39 (68.4)	1.17 (0.56-2.47)	1.12 (0.46-2.73)
Elementary (1-8)	22 (45.8)	26 (54.2)	2.15 (1.02-4.57)	1.39 (0.54-3.62)
High school and above	22 (28.2)	56 (71.8)	1	
Occupation				
Farmer	12 (27.3)	32 (72.7)	1	
Government employee	29 (31.1)	64 (68.8)	1.2 (0.55-2.68)	
Merchant	26 (36.1)	46 (63.9)	1.50 (0.66-3.42)	
Housewife	17 (27.4)	45 (72.6)	1.00 (0.42-2.40)	
Other	2 (77.8)	7 (22.2)	0.76 (0.14-4.20)	
DM duration				
Year < 10	22 (18.5)	97 (81.5)	1	
10-19	51 (36.2)	90 (63.8)	2.50 (1.40-4.45)	
≥20	13 (65.0)	7 (35.0)	8.18 (2.92-22.90)	
BMI (kg/m^2^)				
Normal	12 (66.7)	6 (33.3)	1	
Overweight	55 (26.8)	150 (73.2)	0.10 (0.03-0.33)	0.64 (0.17-2.41)
Obese	19 (33.3)	38 (66.7)	10.36 (0.10-1.27)	1.50 (0.34-6.70)
Type of antidiabetic drug in use				
OH	49 (22.8)	166 (77.2)	0.39 (0.16-0.98)	0.37 (0.13-1.11)
Both OH and insulin	28 (63.6)	16 (36.4)	2.33 (0.81-6.74)	0.98 (0.26-3.66)
Insulin	9 (42.9)	12 (57.1)	1	
HbA1c (%)^C^	7.6 ± 1.2	6.6 ± 0.9	2.04 (1.48-2.83)	1.97 (1.03-3.40)^∗^
Cigarette smoking				
Nonsmoker	49 (22.3)	171 (77.7)	1	
Ex-smoker	23 (59.0)	16 (41.0)	5.02 (2.46-10.23)	4.68 (1.93-11.30)^∗∗^
Current smoker	14 (66.7)	7 (33.3)	6.98 (2.67-18.25)	5.84 (1.79-19.04)^∗∗^
History of HTN self-reported				
Yes	46 (31.5)	100 (68.5)	1.08 (0.65-1.79)	
No	40 (29.9)	94 (70.1)	1	
Current hypertension/high BP				
Yes	64 (40)	96 (60)	2.96 (1.69-5.20)	1.91 (0.88-4.15)
No	22 (18.3)	98 (81.7)	1	

^∗^Statistically significant (*P* value < 0.05). ^∗∗^Statistically highly significant (*P* value < 0.01). AOR: adjusted odds ratio; CI: confidence interval; COR: crude odds ratio. ^C^Continuous.

## Data Availability

The data will be available from the corresponding author upon request.

## Abbreviations

ABI: Ankle-brachial index
BMI: Body mass index
HbA1c: Glycated hemoglobin
HTN: Hypertension
PAD: Peripheral arterial disease
T2DM: Type 2 diabetes mellitus.
